# Dietary macronutrient balance and fungal infection as drivers of spermatophore quality in the mealworm beetle

**DOI:** 10.1016/j.cris.2021.100009

**Published:** 2021-01-16

**Authors:** Alicia Reyes-Ramírez, Maya Rocha-Ortega, Alex Córdoba-Aguilar

**Affiliations:** Departamento de Ecología Evolutiva, Instituto de Ecología, Universidad Nacional Autónoma de México, Apdo. P. 70-275, Circuito Exterior, Ciudad Universitaria, 04510 Coyoacán, Ciudad de México, México

**Keywords:** Carbohydrate, Condition, Diet, Protein, Spermatophore, *Tenebrio molitor*

## Abstract

Males of many insects deliver ejaculates with nutritious substances to females in the form of a spermatophore. Different factors can affect spermatophore quality. We manipulated males’ diet and health to determine the balance of macronutrients deposited in the spermatophores of *Tenebrio molitor* beetles. For diet, we varied the concentration of proteins and carbohydrates, while for health status we used a fungal infection. Males with different condition copulated with unmanipulated females, and spermatophores were extracted to measure the amount of proteins, lipids and carbohydrates. Diet and infection had an effect on the quality of the spermatophore. Diets with high protein and low carbohydrate contents produced spermatophores with higher protein, carbohydrate, and lipid contents. In contrast, diets with little protein and high in carbohydrates led to low quality spermatophores. Infected males produced spermatophores with the highest amount of all three macronutrients. In general, spermatophore content was carbohydrates>proteins>=lipids. The fact that sick males produced richer spermatophores can be explained as a terminal investment strategy. The large investment of carbohydrates may be related to the preparation of spermatozoa in males, and eggs in females.

## Highlights

• We compare the spermatophores produced by healthy and sick males fed with different diets.
• Male diet and health status affect the proteins, lipids and carbohydrates.
• Unexpectedly, sick males produce the richest nutrient spermatophores.
• Sick males may thus use a terminal investment strategy to increase reproductive success.

## 1 Introduction

Traditionally, it was thought that reproductive success of males was limited only by female access (Andersson, 1994) and therefore their investment in gametes should be low (Bateman, 1948; Trivers, 1972). However, we now know that this investment is not trivial (Gwynne, 2008; Lehmann, 2012; Parker and Pizzari, 2010; Vahed, 1998). In fact, by selecting high quality males, females increase their fitness or that of their progeny through indirect and direct benefits they receive from males (Bachmann et al., 2019; Pischedda and Chippindale, 2017; South et al., 2011). While indirect benefits occur via genes that increase the viability or attractiveness of the progeny (Lande, 1981; Zahavi, 1977), direct benefits are material resources that increase females’ fitness, as is the case of, for example, nuptial gifts (e.g. spermatophores) (Levin et al., 2016; Lewis et al., 2014; South et al., 2011).

In many species of insects, the ejaculate is transferred to the female within a spermatophore – a capsule of albuminous material (Chapman et al., 2013). Females maximize their direct fitness benefits when the spermatophore functions as a nuptial gift (that is, when it provides additional energetic resources to females) (Boggs, 2018; Gwynne, 2008; Lewis and South, 2012). The direct benefits gained will vary with the nutritional value/composition of the spermatophore. In particular, spermatophores rich in proteins promote increased fecundity (Karlsson, 1996, 1995; Lehmann, 2012), which explains why these compounds are the most abundant in spermatophores (e.g. Marshall, 1982). However, spermatophores also contain lipids and carbohydrates, though in smaller amounts (Marshall and McNeil, 1989; Stanley-Samuelson and Loher, 1983; Watanabe and Sato, 1993). In the case of lipids, these are also used to produce eggs and synthesize hormones (e.g. Friend, 1958; Levinson, 1962), while carbohydrates are an additional source of energy, though these are less abundant than proteins and lipids (e.g. Mann, 2012; Marshall, 1982). Relatively little is known about the amount of several macronutrients present in the spermatophore, with a few studies that have focused mainly on protein composition (Avila et al., 2011; Lehmann et al., 2018), proteins and lipids (Marshall, 1985) and proteins and carbohydrates (Blanco et al., 2009). Yet, the amount of proteins, lipids and carbohydrates that males assign to spermatophores has been quantified in only two studies (Gerber et al., 1971; Heller et al., 1998).

One of the most important factors determining spermatophore quality is male diet (Cahenzli and Erhardt, 2013; Muller et al., 2015). For example, restricting diet induced males to produce smaller spermatophores in butterflies (Ferkau and Fischer, 2006) and bush-crickets (Gwynne, 1993; Hare and Simmons, 2020). While studies of this kind are conclusive in explaining the role of calories in the diet, we know little about the contribution of specific dietary components (i.e. macronutrients). One way for determining this is the geometric framework (GF) of nutrition, which allows us to examine the effect of specific nutrients on the expression of traits and measures of fitness (Simpson et al., 2018; Simpson and Raubenheimer, 1993; South et al., 2011). The GF is an analytical methodology that is based on the logic of state-space geometry aimed to characterize the key variables responsible for the regulation of nutrients (Raubenheimer et al., 2009). The geometric space may include one or more nutrients, the current and optimal nutritional states of the organism, the efficiency with which such nutrients are used as well as the rates of excretion and/or any other performance measure (Raubenheimer et al., 2009). The closest studies that have determined the effect of diet on spermatophores, are three investigations where GF was used to understand sperm count and viability in cockroaches (Bunning et al., 2015), ants (Dávila and Aron, 2017) and crickets (Ng et al., 2018). The results of these studies are contradictory, however. While in cockroaches a diet restricted in proteins maximized sperm production, in ants restricting the same macronutrient led to a decrease in sperm number. On the other hand, a low protein diet increased sperm viability in crickets but had no effect in cockroaches or ants. Thus, these three studies do not show the same directions in sperm traits (of course, sperm traits [as documented by these three studies] are different to spermatophore quality traits). Thus, it is difficult to make predictions about what to expect in terms of the effect of the balance of proteins, lipids, and carbohydrates in the diet on the general quality of the spermatophore. Given that spermatophore's macronutrient composition involves a high quantity of proteins followed by lipids and carbohydrates (Marshall, 1982), it implies that this should be the natural balance of males’ spermatophore investment.

Diet restrictions are not the only pressures that organisms face in nature. Another type of pressure is infection, where pathogens weaken the state of health and therefore, condition (defined here as the general health and vigor of an organism (Lailvaux and Irschick, 2006). The combined effect of dietary macronutrient and infection has been investigated in several organisms (Lee et al., 2008; Nestel et al., 2016). In this respect, diets with higher protein content led to a more effective response to disease (Alaux et al., 2010; Cotter et al., 2019; Lee et al., 2008; Povey et al., 2014). However, while a diet low in protein will negatively affect an immune response against infections in insects, it would allow to maintain the balance of the proteins>lipids=carbohydrates in the spermatophore, although its investment will be less so than with a protein rich diet.

The flour beetle *Tenebrio molitor* has been used as a study subject to investigate the effects of diet and pathogens on reproduction. Males of this animal transfer a spermatophore to the female during copulation, which increases the number of eggs laid (Drnevich et al., 2001; Worden and Parker, 2001). The spermatophore in this species has been described as a long and narrow tube, enclosed in a scanty gelatinous matrix (Davey, 1960; Jones, 1967), which presents a “plug-shaped” structure at the anterior end (Gadzama and Happ, 1974; Jones, 1967). A recent study did not detect differences in spermatophore quality when varying the proportion of digestible foods in the males’ diets (McConnell and Judge, 2018). However, McConnell & Judge's study did not use synthetic diets, which impedes to assess the effect of a particular macronutrient on the spermatophore. Related to this, the protein:carbohydrate ratio (p:c) has been studied in this animal in terms of life expectancy and fecundity. In an initial study, Rho & Lee (Rho and Lee, 2016) found that while reproductive success should be maximized at a pc ratio of 1:1, an excess of protein reduces life expectancy in both sexes. In a second study on the same animal, lifespan increased when proteins were absent but carbohydrates elevated in healthy males and males infected with the entomopathogenic fungus *Metarhizium robertsii* (Reyes-Ramírez et al., 2019a). Thus, these two studies coincide in their findings that increased protein consumption negatively affects longevity in males, independent of health status.

In this study, we investigated the effects of varying nutritional and health status on spermatophore quality produced by *T. molitor* male adults. A first hypothesis is that nutrition and health status affect both the size and each of the nutrients present in their spermatophore. Accordingly, we predicted that healthy males will produce the largest and protein-rich spermatophores compared to sick males. A second hypothesis is that spermatophores will keep the same variation of the three main nutrients independently of whether diet and health status vary. Thus, we predict that males of all treatments will produce spermatophores with more protein content than carbohydrate and lipid content. A third hypothesis is that nutrition and health status will have an effect on the total amount of the three nutrients, whereby we predict that healthy animals will have more of the three nutrients altogether in the spermatophore compared to sick males.

## 2 Material and methods

### 2.1 Insect maintenance

Individuals were kept in a controlled environment chamber at 70% humidity and 25 ± 2°C (average ± standard deviation) with a 12:12 photoperiod. These conditions were maintained during all life stages of *T. molitor*. We placed 200-300 larvae in plastic containers (30.5 cm diameter x 10.5 cm height) in order to decrease cannibalism (Weaver and McFarlane, 1990). They were fed with wheat germ (Maxilu® brand) and apple slices. Pupae were sexed by observing the eighth abdominal segment (Bhattacharya et al., 1970) and were placed in plastic containers (22.1 cm length x 15.4 cm width x 5.7 cm height). Males and females were separated as soon as they emerged to ensure they were virgins at the time of the experiment. Both sexes were placed individually in plastic containers (4.2 cm diameter x 3.8 cm tall). Females were fed the same diet as when they were larvae and males were fed synthetic diets (see below).

### 2.2 Synthetic diets

To vary the nutritional condition of males only, five synthetic diets were established (Dadd, 1960; Simpson and Abisgold, 1985) that differed in their proportion of proteins to digestible carbohydrates (% dry mass p:c). Proteins consisted of a mix of casein, peptone, and albumin in a 3:1:1 proportion, while carbohydrates were sucrose and dextrin in a 1:1 ratio. The diets were composed of the following proportions: 1) 80% proteins and 0% carbohydrates (p80:c0); 2) 64% proteins and 16 % carbohydrates (p64:c16); 3) 40% proteins and 40% carbohydrates (p40:c40); 4) 16% proteins and 64% carbohydrates (p16:c64); and, 5) 0% proteins and 80% carbohydrates (p0:c80). All diets contained the same proportion (20%) of the following nutrients: Wesson salt mixture (2.4%), cholesterol (0.5%), linoleic acid (0.5%), ascorbic acid (0.3%), and a vitamin mixture (0.2%). To complete the diet, cellulose (16.1%) was added as a non-nutritious agent. The reagents used were all purchased from MilliporeSigma (formerly known as Sigma-Aldrich).

**Fig. 1:**
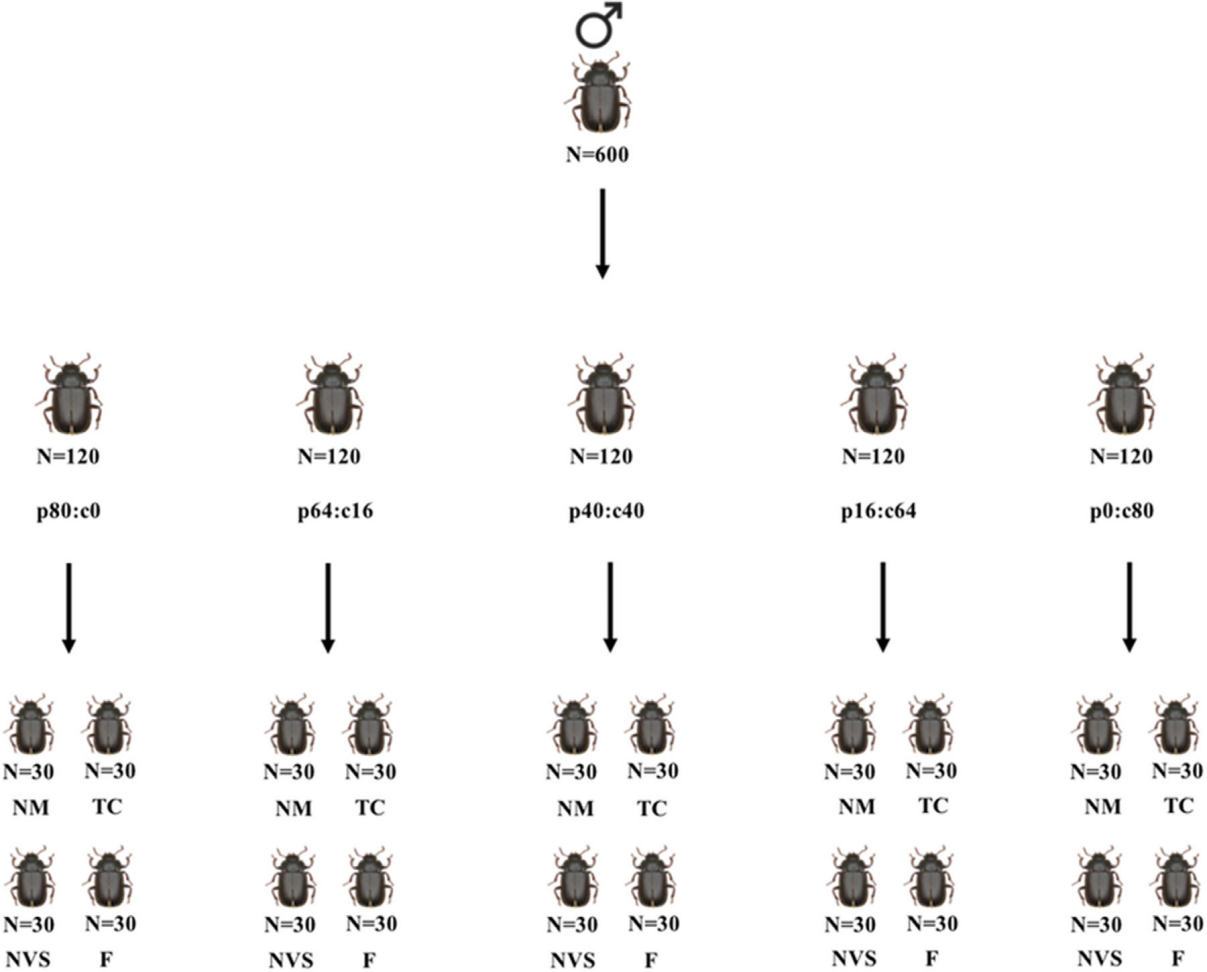
Diagrammatic representation of the experimental design showing the distribution in the five artificial diets (p:c) as well as treatments according to male state of health (NM: non-manipulated, TC: Tween control, NVS: non-viable spores and F: fungus-treated) in *Tenebrio molitor*.

Recently emerged adult males were randomized into one of the five synthetic diets (for graphical illustration of experimental design, see Fig. 1). These diets were provided *ad libitum* in feeders (the previously mentioned plastic containers) in a “food dish” (inverted top of a 1.5 mL Eppendorf tube [9 mm diameter, 5 mm depth]) during the experimental period (day 0 to day 15). The males also had access to water *ad libitum* (20 µl of water per day). The “dishes” were removed and replaced with new ones every other day.

### 2.3 Preparation of the fungus and determination of LD_50_

*Metarhizium robertsii* is a natural pathogen of *T. molitor* (Oliveira et al., 2018) as well as 600 other species of insects (Barelli et al., 2016; Branine et al., 2019; Sasan and Bidochka, 2012), commonly found in soils. The speed of killing depends on the dose, but even under a high dose of conidia, it takes several days after infection to penetrate the cuticle, multiply, intoxicate and finally kill the host (Scholte et al., 2006, 2004). The fungus *M. robertsii* (ARSEF 2134) was obtained from the Agricultural Research Service of the United States Department of Agriculture. Spores were stored in 10% glycerol at -80°C until use. The inoculum was prepared by seeding spores on Sabouraud Dextrose Agar (SDA) plates and then incubating in the dark at 28°C for 15 days. After this period, the conidiophores were harvested by scraping the plates and suspending them in 0.03% Tween 80 solution (hereafter referred to as Tween). The suspension was mixed for 5 minutes in a vortex, then filtered using cotton mesh to separate the conidia from the mycelium. Using the SDA plate technique (Goettel and Inglis, 1997), it was determined that the relative viability of the conidia was above 95%. The LD_50_ (median lethal dose) was obtained from this filtrate. This LD_50_ (3 × 10^5^ conidia/mL) has previously been reported by Reyes‐Ramírez et al. (2019b).

### 2.4 Variation in the state of health due to pathogens in males

To vary health status in males, we used the same males whose nutritional condition was modified (see Fig. 1). Within each diet group, four health status groups were formed. Each group contained 30 mature individuals of 12-15 days of age (Gerber, 1976) named as follows: (i) negative control (unmanipulated individuals); (ii) Tween control, individuals submerged in Tween for 5 seconds; (iii) spore-treated (non-viable spores) individuals submerged for 5 seconds in Tween with *M. robertsii* (ARSEF 2134) spores that had been previously exposed to high temperatures to make them inviable; and (iv) fungus-treated, individuals submerged in Tween with 3 × 10^5^ conidia/mL (LD_50_) of *M. robertsii* for 5 seconds.

### 2.5 Obtaining and dissecting spermatophores

Three days after the males’ health status was modified, pairs were randomly formed using females in good condition and the different groups of males (variation in nutritional and health status). Each pair was placed in plastic containers (4.2 cm diameter x 3.8 cm height) to allow them to copulate (note that individuals were never used more than once). Immediately after completing one copulation, females were removed, frozen dry at −20 °C and refrigerated to later extract the spermatophores (one spermatophore per each female). The spermatophores were gently removed from the bursa copulatrix with forceps and dissecting pins. All dissections were carried out in *Tenebrio* saline buffer (Butz, 1957), which was also used to store them individually in 2 mL Eppendorf tubes. The spermatophores were kept at -20°C until macronutrient measurement.

### 2.6 Protein, lipid and carbohydrate measurement

The spermatophores (n=30 for each diet and health status combination) were transferred to new Eppendorf tubes with 180 µL of aqueous lysis buffer [100 mm KH2PO4, 1 mm dithiothreitol (DTT) and 1 mm ethylenediaminetetraacetic acid (EDTA), pH 7.4], to later be disrupted in a Tissue Lyser-II (Qiagen, Valancia, California) for 30 s at 25 Hz. To quantify the amount of nutrients present in the spermatophores, we used the unified Foray method (Foray et al., 2012). In this method, different solvents are used to sequentially extract the desired components and quantify them using specific colorimetric techniques. Briefly, after agitating, the proteins in the samples were solubilized in a phosphate lysis buffer and quantified with the Bradford method (Bradford, 1976). Bovine serum albumin was used as a standard and absorbance was determined at 595 nm. In the case of total lipids and carbohydrates we used the Van Handel & Day method (Van Handel, 1985; Van Handel and Day, 1988). For lipids, we did a vanillin assay with trioleate glycerol as a standard and measured absorbance at 515 nm. Note that although vanillin assay is considered an accurate technique (Williams et al., 2011), it depends on composition of samples and standards. Also, this technique cannot separate neutral lipids from polar lipids. When using a TAG (triacylglyceride) standard (like trioleate glycerol), this method assumes that all fatty acids originate from TAGs (Williams et al., 2011). Therefore, here we use the vanillin assay under the assumption that there is no change in fatty acid saturation among treatments. For carbohydrates, the anthrone colorimetric method was used with D-glucose as a standard and absorbance determined at 630 nm. All absorbance measurements were done on an absorbance reader (Absorbance Reader ELx800; BioTek Inc., Winooski, Vermont). The concentration of the three macronutrients was calculated taking into account the size (mm^2^) of the spermatophores. The size was obtained from photos analyzed in a publicly available image program (ImageJ).

### 2.7 Statistical analysis

To examine the effect of diet and state of health on spermatophores size, an analysis of variance (ANOVA) was constructed. The spermatophores size (mm^2^) corresponded to the dependent variable, while diet and state of health were the independent variables. Because significant differences were found in state of health and the interaction between diet and health status, we made comparisons between treatments using the post-hoc Least Significant Difference (LSD) test whereby a separate analysis for each group and corresponding interactions are obtained (Engqvist, 2005). Multiple comparisons between each group mean to the control mean were corrected using the Benjamini-Hochberg procedure. In the case of diet, the control was p0:c80, while for state of health was the fungus treatment.

To determine how diet and state of health affected each of the macronutrients (total proteins, lipids and carbohydrates) present in spermatophores, independent multivariate analyses of variance (MANOVAs) fit with Pillai's trace per nutrient, were constructed. For multivariate analysis, Pillai's trace is considered the most robust fit against violation of test assumptions (Scheiner, 1993). A MANOVA was carried out per macronutrient, yielding a total of three separate analyses. In the first model, the amount of protein corresponded to the dependent variable, while diet and state of health were independent variables. In the second model, the amount of lipids corresponded to the dependent variable, while diet and state of health were independent variables. In the third model, the amount of carbohydrates corresponded to the dependent variable, while diet and state of health were entered as independent variables. Following MANOVAs, treatments were compared with an LSD test using the Benjamini-Hochberg procedure, to account for multiple comparisons between each group mean against the control mean. In the case of diet, the control was the p0:c80 diet, while for state of health was the fungus.

Another two-way ANOVA was constructed to test if diet and state of health affect the total nutrients amount present in spermatophores. In the model, the amount of nutrients (proteins + lipids + carbohydrates) correspond to the dependent variable, while diet and state of health were the independent variables. Following statistical significances among independent variables, we made comparisons between control vs treatments using the post-hoc Least Significant Difference test with Benjamini-Hochberg procedure. In the case of diet, the control was the p0:c80 diet, while for state of health was the fungus treatment. All analyses were carried out in R in version 3.4.2 (Team, 2017) with the “emmeans” (Lenth and Lenth, 2018), “multcompView” (Graves et al., 2015) and “car” (Fox and Weisberg, 2019) packages.

## 3 Results

### 3.1 Effect of nutrition and health status on spermatophore size

Spermatophore size (in mm^2^) was not affected by diet the males were fed with (F_4_=1.425, P>0.05), but was affected by their health (F_3_=9.342, P<0.001) and by the interaction between diet and health status (F_12_=2.508, P<0.01). Non-manipulated males fed with the p40:c40 diet and non-manipulated males and those challenged with non-viable spores fed with the p0:c80 diet produced larger spermatophores than the fungus males from the same diets (for all combinations see supplementary material Table S1).

### 3.2 Effect of nutrition and health status on each spermatophore nutrient: protein

The results indicated that diet (F_9_=3.483, P<0.001), health status (F_3_=36.469, P<0.001) and the interaction (F_27_=2.419, P<0.001) had an effect on the amount of total protein in the spermatophore. Individuals fed with the p16:c64 diet had lower protein amount (for all combinations, see supplementary material Table S2).

The health status of the males also affected the amount of proteins in their spermatophores. The spermatophores produced by males treated with the fungus had the highest protein content than the three control groups: non-manipulated, Tween control and spore-treated males (for all combinations see supplementary material Table S3).

### 3.3 Effect of nutrition and health status on each spermatophore nutrient: lipids

The total lipid amount in the spermatophores varied depending on diet (F_9_=40.494, P<0.001), health status (F_3_=56.747, P<0.001) and the interaction between diet and health status (F_27_=7.634, P<0.001). The diets with which males invested the most lipids in spermatophores were p64:c16 and p40:c40, while the p80:c0 and p16:c64, produced the lowest lipid content (for all combinations see supplementary material Table S4).

Likewise, the health status of the males influenced the lipids deposited in the spermatophore. Males treated with fungus produced spermatophores with the highest total lipid amount than the control groups (for all combinations see supplementary material Table S5).

### 3.4 Effect of nutrition and health status on each spermatophore nutrient: carbohydrates

Finally, diet (F_9_=18.063, P<0.001), health status (F_3_=68.364, P<0.001) and the interaction between diet and health status (F_27_=3.234, P<0.001) affected the total carbohydrate content present in the spermatophores. Individuals fed with the p64:c16 and p40:c40 diet invested more carbohydrates in their spermatophores, while those fed with the p80:c0, p16:c64, and p0:c80 diets showed the lowest investment (for all combinations see supplementary material Table S6).

Health status also had an effect. Fungus-treated males again produced the spermatophores with the highest carbohydrate content than the non-manipulated, Tween control and spore-treated males (for all combinations see supplementary material Table S7).

### 3.5 Effect of nutrition and health status on the balance of proteins/lipids/carbohydrates in spermatophores

**Fig. 2:**
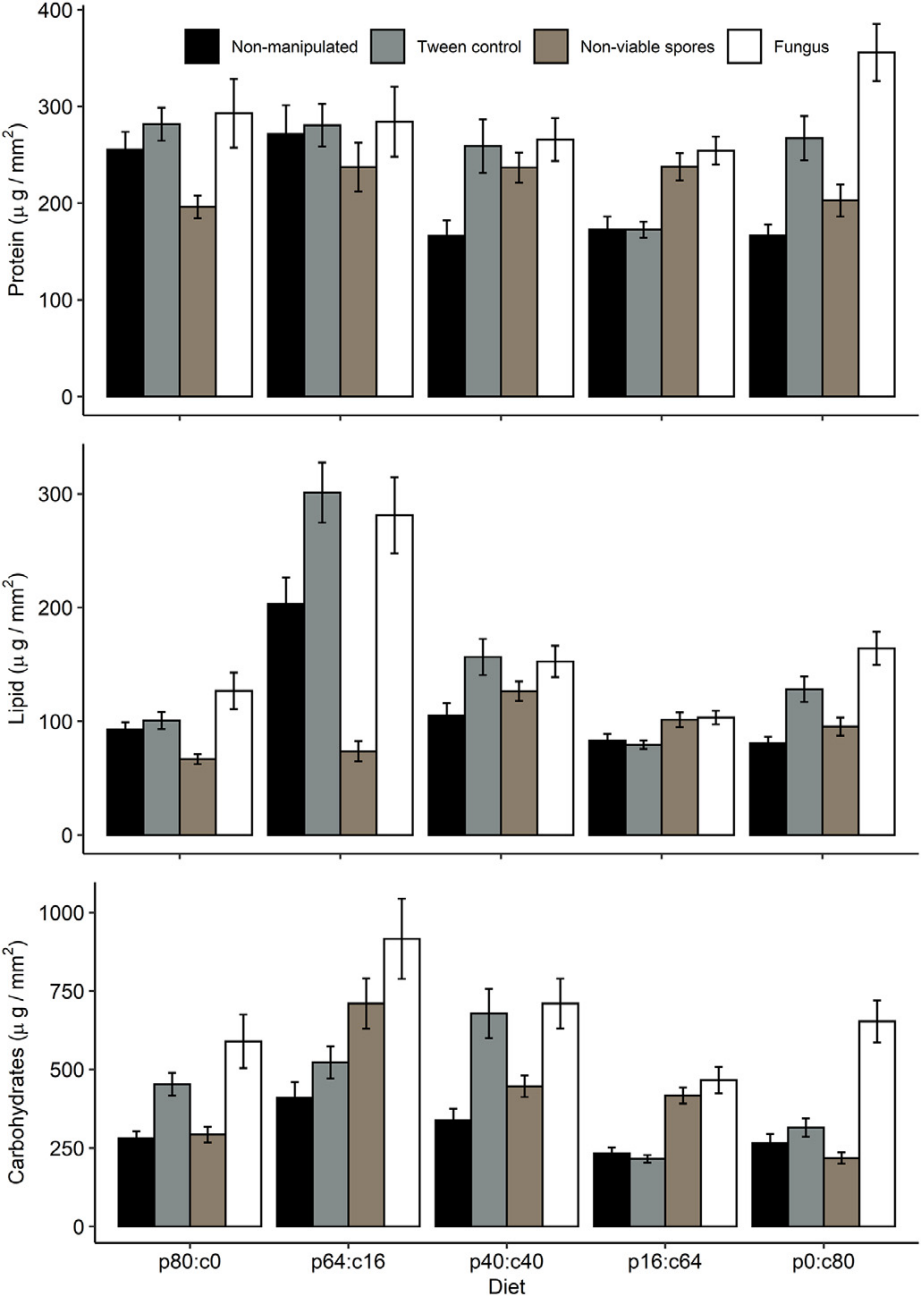
Changes in spermatophore nutrient content (carbohydrate, lipid and protein; mean ±SE) following changing ratios of protein:carbohydrate (p:c) and experimental manipulation of status of health in *Tenebrio molitor* males.

In general, visual inspection of the proportion of the three components (see supplementary material Fig. 1) suggested the following. Most treatments yielded an order of c>p>l in spermatophores (see Table 1). Some exceptions to this were the negative control males and spore-treated males under the 80:0 diet, in which the balance was protein=carbohydrate>lipid, and the Tween control and fungus-treated males on the 64:16 diet and the negative control on the 40:40 diet, in which the balance was carbohydrate>protein=lipid (see also Fig. 2).

**Table 1:** Summary of mean macronutrients amount (µg/mm^2^) presents in spermatophores according to the diet and health status.

Health status	Dietary P:C ratio	Mean macronutrients amount (± SE)				
		N	Proteins	Lipids	Carbohydrates	Total
Non-manipulated	80:0	30	255.4 ± 18.2	92.7 ± 6.5	280.9 ± 22.8	629.1 ± 45.7
	64:16	30	271.4 ± 29.9	203.3 ± 23.08	410.3 ± 50.2	885.1 ± 100.4
	40:40	30	166.2 ± 15.9	105.1 ± 10.6	338.3 ± 37	609.7 ± 59.2
	16:64	30	172.5 ± 13.4	83.02 ± 6.01	232.9 ± 18.6	488.4 ± 37.2
	0:80	30	166.6 ± 11.2	80.7 ± 5.5	265.1 ± 29.3	512.5 ± 43.6
Tween control	80:0	30	281.6 ± 17.06	100.6 ± 7.4	453.5 ± 36.06	835.8 ± 57.7
	64:16	30	280.5 ± 22.03	301.07 ± 26.4	522.6 ± 51.4	1104.2 ± 89.4
	40:40	30	259.1 ± 27.6	156.5 ± 15.9	678.4 ± 78.5	1094 ± 120.1
	16:64	30	172.6 ± 8.2	79.4 ± 3.7	215.5 ± 12.1	467.6 ± 21.7
	0:80	30	267.05 ± 22.9	128.1 ± 11.1	315 ± 29.1	710.2 ± 61.7
Non-viable spores	80:0	30	196.1 ± 11.7	66.7 ± 4.3	292.5 ± 25.1	555.4 ± 39.4
	64:16	30	237.1 ± 25.2	73.6 ± 8.9	709.8 ± 79.5	1020 ± 111.8
	40:40	30	236.7 ± 15.7	126.4 ± 8.6	446.9 ± 34.5	810.1 ± 54.2
	16:64	30	237.5 ± 14.2	101.3 ± 6.4	417.2 ± 25.3	756.1 ± 41.8
	0:80	30	202.6 ± 16.6	95.4 ± 7.9	218 ± 17.7	516.1 ± 39.9
Fungus	80:0	30	292.8 ± 35.5	126.7 ± 16.05	589.9 ± 85.1	1009.6 ± 134
	64:16	30	284.2 ± 36.1	281.01 ± 33.3	916.1 ± 127.8	1481 ± 195.6
	40:40	30	265.7 ± 22.1	152.6 ± 13.8	710.3 ± 79.01	1128.6 ± 110
	16:64	30	254.3 ± 14.4	103.3 ± 5.9	466.5 ± 42.01	824.1 ± 59.9
	0:80	30	355.8 ± 29.4	164.2 ± 14.5	652.9 ± 66.9	1173 ± 104.6

Given the trends described above, the proportion assigned to each macronutrient also showed notable differences (again for visual inspection see Supplementary material Fig. 1). For example, carbohydrates were considerably higher than proteins and lipids in most treatments, especially in fungus-treated males and, to a lesser degree, Tween controls. These differences, however, were not as dramatic in the negative control or spore-treated males.

### 3.6 Effect of nutrition and health status on the total amount of nutrients

The results indicated that diet (F_4_=19.18, P<0.001), health status (F_3_=30.14, P<0.001), and the interaction between diet and health status (F_12_=2.23, P<0.01), had an effect on the total amount of nutrients present in the spermatophore. Non-manipulated individuals, meaning those treated with non-viable spores and fungus males, produced the spermatophores with the highest amount of nutrients when fed with the p64:c16 diet. Tween control males presented the highest amount of nutrients in the p64:c16 and p40:c40 diets (for all combinations, see supplementary material Table S8).

The health status of the males also affected the total amount of nutrients in their spermatophores. Spermatophores produced by males treated with the fungus had the highest content of nutrients, followed by Tween control, spore-treated males, and finally non-manipulated males (for all combinations see supplementary material Table S9).

## 4 Discussion

As has been corroborated in other studies where animals’ condition is manipulated to observe the effects on the spermatophore (e.g. Duplouy et al., 2018; Ferkau and Fischer, 2006; Kelly and Gwynne, 2016), in *T. molitor* the macronutrient composition of the spermatophore was sensitive to changes in males’ diet and health status (even when our vanillin assay implied some assumptions). Spermatophore size was not affected by diet but it was by health status. This condition-dependence is expected for energetically demanding characteristics (Kotiaho, 2001; Macartney et al., 2018; Pitnick et al., 2009). The case of the spermatophore may be very costly both in quality (Duplouy et al., 2018; Wagner, 2005; Wiklund and Kaitala, 1995) and quantity (del Castillo and Gwynne, 2007; Kerr et al., 2010; Lehmann and Lehmann, 2009). In the case of diet, we found that in general the p64:c16 ratio was the diet that led males to invest more in the three macronutrients, while the inverse diet, p16:c64, reduced levels. In this way, it can be said that the ideal diet to produce a robust spermatophore is p64:c16, though this would need to be corroborated with a study of reproductive success.

In fact, it is common to see a lack of adaptive sense in the effect of diet on ejaculate traits (e.g. Bunning et al., 2015; Dávila and Aron, 2017; Ng et al., 2018). The most similar study is one in *Drosophila melanogaster.* In this animal egg production is depressed in flies that are fed a diet without proteins and they laid the most eggs when fed a high protein diet, in a proportion of 4:1 (Lee, 2015), similar to our p64:c16 diet. This suggests that protein is essential for offspring survival. However, it should be noted that that such ideal diet is not optimal for the survival of the male, since protein ingestion reduces lifespan (Reyes-Ramírez et al., 2019a; Rho and Lee, 2016). Perhaps the consumption of protein by adults serves to resolve a trade-off between the demands of the spermatophore and what the animal needs to survive. Findings were similar in the cricket *Teleogryllus oceanicus*, where protein consumption similarly decreased survival but increased the song used for courtship and the production of cuticular hydrocarbons (Ng et al., 2018). Like in *T. molitor*, this would assume a trade-off between survival and reproductive traits. In the case of health status, male *T. molitor* infected with the fungus invariably deposited more of all three macronutrients than the other groups. This investment was again most clear under the p64:c16 diet (and considerably less in the p16:c64 diet), which would be expected if it is ideal. In *T. molitor*, protein ingestion does not increase the probability of surviving an attack by *M. robertsii* (Reyes-Ramírez et al., 2019a) but protein is necessary for the spermatophore. It is striking that individuals fed on the diet that did not have any protein intake (p0:c80) did not differ in the content they invested of this nutrient in their spermatophores. Furthermore, we must remember that nutritional variation in this study occurred in the adult stage. So, it is possible that the reserves that accumulated during the larval stage influence. Moreover, in another study it was found that in the p0:c80 diet, both healthy and sick males increased their life expectancy (Reyes-Ramírez et al., 2019a). In this case, by decreasing the risk of dying, males could be allocating their protein reserves towards the production of spermatophores.

Why do sick males invest more in the spermatophore compared to unmanipulated males? One explanation is what is known as terminal investment, where individuals whose survival is at risk invest more in reproduction to maximize their reproductive opportunities (Clutton-Brock, 1984; Duffield et al., 2017; Williams, 1966). In the case of *T. molitor*, the traits that will yield the most direct benefits are the spermatophores. There are several sources of evidence supporting terminal investment in this animal. The first has to do with male pre-copulatory attractiveness: males challenged with inert materials (Kivleniece et al., 2010; Krams et al., 2014; Nielsen and Holman, 2012; Sadd et al., 2006) as well as males infected with the same fungus used in this experiment, *M. robertsii* (Reyes‐Ramírez et al., 2019b), were more successful in attracting females via pheromones. The second source has to do with post-copulatory success: males infected with pathogens provided more proteins in their spermatophores (Hurd and Ardin, 2003), which is similar to what we found here. Paradoxically, increased investment in the spermatophore did not lead to higher reproductive success in *T. molitor* as males infected with *M. robertsii* gave rise to fewer eggs, with lower hatching success and lower lipid content (Reyes‐Ramírez et al., 2019b). Thus, it may be that females are attracted to males that are sick and have a higher macronutrient content in their spermatophores but then penalize them with a lower egg number and quality. This supposes that females recognize males that are carrying out terminal investment. Interestingly, females of this species benefit substantially by using the spermatophores from several males to increase their reproductive success (Drnevich et al., 2001; Worden and Parker, 2001). This ability to “collect” spermatophores as an additional nutrient source but discard the sperm of terminally investing males would imply a large degree of reproductive control by females of this species.

Contrary to our expectation, the nutrient balance of the spermatophore was almost always carbohydrates > proteins > lipids. The protein contribution is expected to be considerably higher to promote increase egg production (Mann, 2012; Marshall, 1982; Murphy and Krupke, 2011). However, an alternative explanation for our results is that carbohydrates play a role in sperm success. In this respect, it has been shown that the seminal fluid of several species of insects generally contains a mixture of short chain carbohydrates or sugars such as fructose, glucose and trehalose (Yaginuma and Happ, 1988). Indeed, in *T. molitor* the presence of glucose and trehalose has been reported in both the bean-shaped accessory glands and in the spermatophore, with trehalose being the more abundant (Gadzama and Happ, 1974). In vitro studies demonstrated that the activity of the trehalase enzyme was not required for the evacuation of the spermatophore, suggesting that the trehalase-trehalose system plays a physiological role in sperm activation and viability (Yaginuma et al., 1996). Subsequent studies should test the effect of trehalose on the viability and activation of sperm, to understand the role of carbohydrate abundance in the spermatophores of this species.

Our study shows that spermatophore characteristics are dynamic, influenced by the male's condition. While the adaptive function of the changes to macronutrient composition we observed is not clear, they are expected to have differential effects on reproduction. One key topic would be to measure food intake to see how animals respond to infection. For example, given a pathogen attack, animals may either show a reduction (to reduce the ingested food that would be available for the pathogen) (Adamo et al., 2007; Kyriazakis et al., 1998) or increase (to provide energetic resources to their immune system) (Ponton et al., 2013; Slansky, 1986) in food intake. Our study should be replicated in other systems where the spermatophore plays a key role in the evolution of mating systems. Examples include the cases of butterflies (Arnqvist and Nilsson, 2000; Cardoso and Silva, 2015; Oberhauser, 1988) and crickets (Burpee and Sakaluk, 1993; Kerr et al., 2010; Sturm, 2014) where experiments have not included analysis of macronutrients but whose effects can be key to reproductive success. In the case of *T. molitor*, the differential effects of diet probably combine with females’ parental investment decisions.

To conclude, our findings show that spermatophores are sensitive to changes in nutrition and health status of males. Although healthy individuals produced the largest spermatophores, sick males surprisingly gave rise to the richest nutrient-based spermatophores. At the interspecific level and unlike other insects, *T. molitor* produce spermatophores whose carbohydrate content is the richest, followed by proteins and lipids. The benefits females and offspring gain from this nutrient composition must be the next step to investigate.

## Data availability

Data are available along with this paper as supplementary material

## Author contributions

ARR and ACA conceived the ideas and designed methodology; ARR made the experiments; MRO analyzed the data; ARR and ACA wrote the manuscript.

## Declaration of Competing Interest

The authors declare that they have no known competing financial interests or personal relationships that could have appeared to influence the work reported in this paper.

## Appendix Supplementary materials

Image, application 1

Image, application 2

Image, application 3

## References

[bib0001] Adamo S.A., Fidler T.L., Forestell C.A. (2007). Illness-induced anorexia and its possible function in the caterpillar, Manduca sexta. Brain. Behav. Immun..

[bib0002] Alaux C., Ducloz F., Crauser D., Le Conte Y. (2010). Diet effects on honeybee immunocompetence. Biol. Lett..

[bib0003] Andersson M.B. (1994).

[bib0004] Arnqvist G., Nilsson T. (2000). The evolution of polyandry: multiple mating and female fitness in insects. Anim. Behav..

[bib0005] Avila F.W., Sirot L.K., LaFlamme B.A., Rubinstein C.D., Wolfner M.F. (2011). Insect seminal fluid proteins: identification and function. Annu. Rev. Entomol..

[bib0006] Bachmann G.E., Devescovi F., Nussenbaum A.L., Milla F.H., Shelly T.E., Cladera J.L., Fernández P.C., Vera M.T., Segura D.F. (2019). Mate choice confers direct benefits to females of Anastrepha fraterculus (Diptera: Tephritidae). PLoS One.

[bib0007] Barelli L., Moonjely S., Behie S.W., Bidochka M.J. (2016). Fungi with multifunctional lifestyles: endophytic insect pathogenic fungi. Plant Mol. Biol..

[bib0008] Bateman A.J. (1948). Intra-sexual selection in Drosophila. Hered. Edinb..

[bib0009] Bhattacharya A.K., Ameel J.J., Waldbauer G.P. (1970). A method for sexing living pupal and adult yellow mealworms. Ann. Entomol. Soc. Am..

[bib0010] Blanco C.A., Rojas M.G., Groot A.T., Morales-Ramos J., Abel C.A. (2009). Size and chemical composition of Heliothis virescens (Lepidoptera: Noctuidae) spermatophores. Ann. Entomol. Soc. Am..

[bib0011] Boggs C.L. (2018). Insect Reproduction.

[bib0012] Bradford M.M. (1976). A rapid and sensitive method for the quantitation of microgram quantities of protein utilizing the principle of protein-dye binding. Anal. Biochem..

[bib0013] Branine M., Bazzicalupo A., Branco S. (2019). Biology and applications of endophytic insect-pathogenic fungi. PLoS Pathog..

[bib0014] Bunning H., Rapkin J., Belcher L., Archer C.R., Jensen K., Hunt J. (2015). Protein and carbohydrate intake influence sperm number and fertility in male cockroaches, but not sperm viability. Proc. R. Soc. B Biol. Sci..

[bib0015] Burpee D.M., Sakaluk S.K. (1993). Repeated matings offset costs of reproduction in female crickets. Evol. Ecol..

[bib0016] Butz A. (1957). Effects of sodium, potassium, and calcium ions on the isolated heart of the mealworm, Tenebrio molitor L. J. N.Y. Entomol. Soc..

[bib0017] Cahenzli F., Erhardt A. (2013). Nectar amino acids enhance reproduction in male butterflies. Oecologia.

[bib0018] Cardoso M.Z., Silva E.S. (2015). Spermatophore quality and production in two Heliconius butterflies with contrasting mating systems. J. Insect Behav..

[bib0019] Chapman R.F., Simpson S.J., Douglas A.E. (2013).

[bib0020] Clutton-Brock T.H. (1984). Reproductive effort and terminal investment in iteroparous animals. Am. Nat..

[bib0021] Cotter S.C., Reavey C.E., Tummala Y., Randall J.L., Holdbrook R., Ponton F., Simpson S.J., Smith J.A., Wilson K. (2019). Diet modulates the relationship between immune gene expression and functional immune responses. Insect Biochem. Mol. Biol..

[bib0022] Dadd R.H. (1960). The nutritional requirements of locusts—I development of synthetic diets and lipid requirements. J. Insect Physiol..

[bib0023] Davey K.G. (1960). Proceedings of the Royal Entomological Society of London. Series A, General Entomology.

[bib0024] Dávila F., Aron S. (2017). Protein restriction affects sperm number but not sperm viability in male ants. J. Insect Physiol..

[bib0025] del Castillo R.C., Gwynne D.T. (2007). Increase in song frequency decreases spermatophore size: correlative evidence of a macroevolutionary trade-off in katydids (Orthoptera: Tettigoniidae). J. Evol. Biol..

[bib0026] Drnevich J.M., Papke R.S., Rauser C.L., Rutowski R.L. (2001). Material benefits from multiple mating in female mealworm beetles (Tenebrio molitor L.). J. Insect Behav..

[bib0027] Duffield K.R., Bowers E.K., Sakaluk S.K., Sadd B.M. (2017). A dynamic threshold model for terminal investment. Behav. Ecol. Sociobiol..

[bib0028] Duplouy A., Woestmann L., Gallego Zamorano J., Saastamoinen M. (2018). Impact of male condition on his spermatophore and consequences for female reproductive performance in the Glanville fritillary butterfly. Insect Sci..

[bib0029] Engqvist L. (2005). The mistreatment of covariate interaction terms in linear model analyses of behavioural and evolutionary ecology studies. Anim. Behav..

[bib0030] Ferkau C., Fischer K. (2006). Costs of reproduction in male Bicyclus anynana and Pieris napi butterflies: effects of mating history and food limitation. Ethology.

[bib0031] Foray V., PELISSON P., BEL-VENNER M., Desouhant E., Venner S., Menu F., Giron D., Rey B. (2012). A handbook for uncovering the complete energetic budget in insects: the van Handel's method (1985) revisited. Physiol. Entomol..

[bib0032] Fox J., Weisberg S. (2019).

[bib0033] Friend W.G. (1958). Nutritional requirements of phytophagous insects. Annu. Rev. Entomol..

[bib0034] Gadzama N.M., Happ G.M. (1974). The structure and evacuation of the spermatophore of Tenebrio molitor L.(Coleoptera: Tenebrionidae). Tissue Cell.

[bib0035] Gerber G.H. (1976). Reproductive behaviour and physiology of Tenebrio molitor (Coleoptera: Tenebrionidae). III. Histogenetic changes in the internal genitalia, mesenteron, and cuticle during sexual maturation. Can. J. Zool..

[bib0036] Gerber G.H., Church N.S., Rempel J.G. (1971). The structure, formation, histochemistry, fate, and functions of the spermatophore of Lytta nuttalli Say (Coleoptera: Meloidae). Can. J. Zool..

[bib0037] Goettel M.S., Inglis G.D. (1997). Manual of Techniques in Insect Pathology.

[bib0038] Graves S., Piepho H.-P., Selzer M.L. (2015).

[bib0039] Gwynne D.T. (1993). Food quality controls sexual selection in Mormon crickets by altering male mating investment. Ecology.

[bib0040] Gwynne D.T. (2008). Sexual conflict over nuptial gifts in insects. Annu. Rev. Entomol..

[bib0041] Hare R.M., Simmons L.W. (2020). Advances in the Study of Behavior.

[bib0042] Heller K.G., Faltin S., Fleischmann P., Helversen O.V (1998). The chemical composition of the spermatophore in some species of phaneropterid bushcrickets (Orthoptera: Tettigonioidea). J. Insect Physiol..

[bib0043] Hurd H., Ardin R. (2003). Infection increases the value of nuptial gifts, and hence male reproductive success, in the Hymenolepis diminuta-Tenebrio molitor association. Proc. R. Soc. Lond. Ser. B Biol. Sci..

[bib0044] Jones J.M. (1967).

[bib0045] Karlsson B. (1995). Resource allocation and mating systems in butterflies. Evol. N.Y..

[bib0046] Karlsson B. (1996). Male reproductive reserves in relation to mating system in butterflies: a comparative study. Proc. R. Soc. Lond. Ser. B Biol. Sci..

[bib0047] Kelly C.D., Gwynne D.T. (2016). The effect of condition on mate searching speed and copulation frequency in the Cook Strait giant weta. Behav. Ecol. Sociobiol..

[bib0048] Kerr A.M., Gershman S.N., Sakaluk S.K. (2010). Experimentally induced spermatophore production and immune responses reveal a trade-off in crickets. Behav. Ecol..

[bib0049] Kivleniece I., Krams I., Daukšte J., Krama T., Rantala M.J. (2010). Sexual attractiveness of immune-challenged male mealworm beetles suggests terminal investment in reproduction. Anim. Behav..

[bib0050] Kotiaho J.S. (2001). Costs of sexual traits: a mismatch between theoretical considerations and empirical evidence. Biol. Rev..

[bib0051] Krams I.A., Krama T., Moore F.R., Kivleniece I., Kuusik A., Freeberg T.M., Mänd R., Rantala M.J., Daukšte J., Mänd M. (2014). Male mealworm beetles increase resting metabolic rate under terminal investment. J. Evol. Biol..

[bib0052] Kyriazakis I., Tolkamp B.J., Hutchings M.R. (1998). Towards a functional explanation for the occurrence of anorexia during parasitic infections. Anim. Behav..

[bib0053] Lailvaux S.P., Irschick D.J. (2006). A functional perspective on sexual selection: insights and future prospects. Anim. Behav..

[bib0054] Lande R. (1981). Models of speciation by sexual selection on polygenic traits. Proc. Natl. Acad. Sci..

[bib0055] Lee K.P. (2015). Dietary protein: carbohydrate balance is a critical modulator of lifespan and reproduction in Drosophila melanogaster: a test using a chemically defined diet. J. Insect Physiol..

[bib0056] Lee K.P., Simpson S.J., Wilson K. (2008). Dietary protein-quality influences melanization and immune function in an insect. Funct. Ecol..

[bib0057] Lehmann G.U.C. (2012). Weighing costs and benefits of mating in bushcrickets (Insecta: Orthoptera: Tettigoniidae), with an emphasis on nuptial gifts, protandry and mate density. Front. Zool..

[bib0058] Lehmann G.U.C., Lehmann A.W. (2009). Condition-dependent spermatophore size is correlated with male's age in a bushcricket (Orthoptera: Phaneropteridae). Biol. J. Linn. Soc..

[bib0059] Lehmann G.U.C., Lehmann K., Neumann B., Lehmann A.W., Scheler C., Jungblut P.R. (2018). Protein analysis of the spermatophore reveals diverse compositions in both the ampulla and the spermatophylax in a bushcricket. Physiol. Entomol..

[bib0060] Lenth R., Lenth M.R. (2018). Package ‘lsmeans.’. Am. Stat..

[bib0061] Levin E., Mitra C., Davidowitz G. (2016). Fed males increase oviposition in female hawkmoths via non-nutritive direct benefits. Anim. Behav..

[bib0062] Levinson Z.H. (1962). The function of dietary sterols in phytophagous insects. J. Insect Physiol..

[bib0063] Lewis S., South A. (2012). Advances in the Study of Behavior.

[bib0064] Lewis S.M., Vahed K., Koene J.M., Engqvist L., Bussiere L.F., Perry J.C., Gwynne D., Lehmann G.U.C. (2014). Emerging issues in the evolution of animal nuptial gifts. Biol. Lett..

[bib0066] Macartney E.L., Crean A.J., Bonduriansky R. (2018). Epigenetic paternal effects as costly, condition-dependent traits. Hered. Edinb..

[bib0067] Mann T. (2012).

[bib0068] Marshall L.D. (1982). Male nutrient investment in the Lepidoptera: what nutrients should males invest?. Am. Nat..

[bib0069] Marshall L.D. (1985). Protein and lipid composition of Colias philodice and C. eurytheme spermatophores and their changes over time (Pieridae). J. Res. Lepid..

[bib0070] Marshall L.D., McNeil J.N. (1989). Spermatophore mass as an estimate of male nutrient investment: a closer look in Pseudaletia unipuncta (Haworth)(Lepidoptera: Noctuidae). Funct. Ecol..

[bib0071] McConnell M.W., Judge K.A. (2018). Body size and lifespan are condition dependent in the mealworm beetle, Tenebrio molitor, but not sexually selected traits. Behav. Ecol. Sociobiol..

[bib0072] Muller K., Thiéry D., Moret Y., Moreau J. (2015). Male larval nutrition affects adult reproductive success in wild European grapevine moth (Lobesia botrana). Behav. Ecol. Sociobiol..

[bib0073] Murphy A.F., Krupke C.H. (2011). Mating success and spermatophore composition in western corn rootworm (Coleoptera: Chrysomelidae). Environ. Entomol..

[bib0074] Nestel D., Papadopoulos N.T., Pascacio-Villafán C., Righini N., Altuzar-Molina A.R., Aluja M. (2016). Resource allocation and compensation during development in holometabolous insects. J. Insect Physiol..

[bib0075] Ng S.H., Simpson S.J., Simmons L.W. (2018). Macronutrients and micronutrients drive trade-offs between male pre-and postmating sexual traits. Funct. Ecol..

[bib0076] Nielsen M.L., Holman L. (2012). Terminal investment in multiple sexual signals: immune-challenged males produce more attractive pheromones. Funct. Ecol..

[bib0077] Oberhauser K.S. (1988). Male monarch butterfly spermatophore mass and mating strategies. Anim. Behav..

[bib0078] Oliveira A.S., Braga G.U.L., Rangel D.E.N. (2018). Metarhizium robertsii illuminated during mycelial growth produces conidia with increased germination speed and virulence. Fungal Biol..

[bib0079] Parker G.A., Pizzari T. (2010). Sperm competition and ejaculate economics. Biol. Rev..

[bib0080] Pischedda A., Chippindale A.K. (2017). Direct benefits of choosing a high-fitness mate can offset the indirect costs associated with intralocus sexual conflict. Evol. N.Y..

[bib0081] Pitnick S., Hosken D.J., Birkhead T.R. (2009). Sperm Biology.

[bib0082] Ponton F., Wilson K., Holmes A.J., Cotter S.C., Raubenheimer D., Simpson S.J. (2013). Integrating nutrition and immunology: a new frontier. J. Insect Physiol..

[bib0083] Povey S., Cotter S.C., Simpson S.J., Wilson K. (2014). Dynamics of macronutrient self-medication and illness-induced anorexia in virally infected insects. J. Anim. Ecol..

[bib0084] Raubenheimer D., Simpson S.J., Mayntz D. (2009). Nutrition, ecology and nutritional ecology: toward an integrated framework. Funct. Ecol..

[bib0085] Reyes-Ramírez A., Rocha-Ortega M., Córdoba-Aguilar A. (2019). Female preferences when female condition and male ornament expression vary. Biol. J. Linn. Soc..

[bib0086] Reyes-Ramírez A., Enríquez-Vara J.N., Rocha-Ortega M., Téllez-García A., Córdoba-Aguilar A. (2019). Female choice for sick males over healthy males: Consequences for offspring. Ethology.

[bib0087] Rho M.S., Lee K.P. (2016). Balanced intake of protein and carbohydrate maximizes lifetime reproductive success in the mealworm beetle, Tenebrio molitor (Coleoptera: Tenebrionidae). J. Insect Physiol..

[bib0088] Sadd B., Holman L., Armitage H., Lock F., Marland R., Siva-Jothy M.T. (2006). Modulation of sexual signalling by immune challenged male mealworm beetles (Tenebrio molitor, L.): evidence for terminal investment and dishonesty. J. Evol. Biol..

[bib0089] Sasan R.K., Bidochka M.J. (2012). The insect-pathogenic fungus Metarhizium robertsii (Clavicipitaceae) is also an endophyte that stimulates plant root development. Am. J. Bot..

[bib0090] Scheiner S.M. (1993). MANOVA: multiple response variables and multispecies interactions. Des. Anal. Ecol. Exp..

[bib0091] Scholte E.-J., Knols B.G.J., Samson R.A., Takken W. (2004). Entomopathogenic fungi for mosquito control: a review. J. Insect Sci..

[bib0092] Scholte E.-J., Knols B.G.J., Takken W. (2006). Infection of the malaria mosquito Anopheles gambiae with the entomopathogenic fungus Metarhizium anisopliae reduces blood feeding and fecundity. J. Invertebr. Pathol..

[bib0093] Simpson S.J., Abisgold J.D. (1985). Compensation by locusts for changes in dietary nutrients: behavioural mechanisms. Physiol. Entomol..

[bib0094] Simpson S.J., Raubenheimer D. (1993). A multi-level analysis of feeding behaviour: the geometry of nutritional decisions. Philos. Trans. R. Soc. Lond. Ser. B Biol. Sci..

[bib0095] Simpson S.J., Ribeiro C., González-Tokman D., Córdoba-Aguilar A., González-Tokman D., González-Santoyo I. (2018). Insect Behavior: From Mechanisms to Ecological and Evolutionary Consequences.

[bib0096] Slansky F. (1986). Nutritional ecology of endoparasitic insects and their hosts: an overview. J. Insect Physiol..

[bib0097] South S.H., House C.M., Moore A.J., Simpson S.J., Hunt J. (2011). Male cockroaches prefer a high carbohydrate diet that makes them more attractive to females: implications for the study of condition dependence. Evol. Int. J. Org. Evol..

[bib0098] Stanley-Samuelson D.W., Loher W. (1983). Arachidonic and other long-chain polyunsaturated fatty acids in spermatophores and spermathecae of Teleogryllus commodus: significance in prostaglandin-mediated reproductive behaviour. J. Insect Physiol..

[bib0099] Sturm R. (2014). Comparison of sperm number, spermatophore size, and body size in four cricket species. J. Orthoptera Res..

[bib0102] Team, R.C., R: A language and environment for statistical computing. R Found. Stat. Comput. Vienna, Austria https//www.R-project.org.

[bib0104] Trivers R.L., Campbell B. (1972).

[bib0105] Vahed K. (1998). The function of nuptial feeding in insects: a review of empirical studies. Biol. Rev..

[bib0106] Van Handel E. (1985). Rapid determination of total lipids in mosquitoes. J. Am. Mosq. Control Assoc..

[bib0107] Van Handel E., Day J.F. (1988). Assay of lipids, glycogen and sugars in individual mosquitoes: correlations with wing length in field-collected Aedes vexans. J. Am. Mosq. Control Assoc..

[bib0108] Wagner W.E. (2005). Male field crickets that provide reproductive benefits to females incur higher costs. Ecol. Entomol..

[bib0109] Watanabe M., Sato K. (1993). A spermatophore structured in the bursa copulatrix of the small white Pieris rapae (Lepidoptera, Pieridae) during copulation, and its sugar content. J. Res. Lepid.

[bib0110] Weaver D.K., McFarlane J.E. (1990). The effect of larval density on growth and development of Tenebrio molitor. J. Insect Physiol..

[bib0111] Wiklund C., Kaitala A. (1995). Sexual selection for large male size in a polyandrous butterfly: the effect of body size on male versus female reproductive success in Pieris napi. Behav. Ecol..

[bib0112] Williams C.M., Thomas R.H., MacMillan H.A., Marshall K.E., Sinclair B.J. (2011). Triacylglyceride measurement in small quantities of homogenised insect tissue: comparisons and caveats. J. Insect Physiol..

[bib0113] Williams G.C. (1966). Natural selection, the costs of reproduction, and a refinement of Lack's principle. Am. Nat..

[bib0114] Worden B.D., Parker P.G. (2001). Polyandry in grain beetles, Tenebrio molitor, leads to greater reproductive success: material or genetic benefits?. Behav. Ecol..

[bib0115] Yaginuma T., Happ G.M. (1988). Trehalase from the bean-shaped accessory glands and the spermatophore of the male mealworm beetle, Tenebrio molitor. J. Comp. Physiol. B.

[bib0116] Yaginuma T., Mizuno T., Mizuno C., Ikeda M., Wada T., Hattori K., Yamashita O., Happ G.M. (1996). Trehalase in the spermatophore from the bean-shaped accessory gland of the male mealworm beetle, Tenebrio molitor: purification, kinetic properties and localization of the enzyme. J. Comp. Physiol. B.

[bib0117] Zahavi A. (1977). The cost of honesty (further remarks on the handicap principle). J. Theor. Biol..

