# Supplementary material for: Dietary macronutrient balance and fungal infection as drivers of spermatophore quality in the mealworm beetle
Source: Curr Res Insect Sci. 2021 Jan 16;1:100009. doi: 10.1016/j.cris.2021.100009 (PMC9387488; doi:10.1016/j.cris.2021.100009)
Supplement: Supplementary file 1 [file mmc1.docx]

Supplementary material Figure 1. Visual comparisons of relative carbohydrate, lipid and protein allocation to spermatophore following changing ratios of protein:carbohydrate (p:c) in the male diet and manipulation of health status (fungus: fungal challenged males; and controls: Tween control, non-viable spores, and non-manipulated) in *Tenebrio molitor* males.

Table S1 Comparisons of state of health treatment and its effect on spermatophores size of *T. molitor* males according to the Least Significant Difference post-hoc test.

| Diet | State of health | Estimate | *t* | P |
| --- | --- | --- | --- | --- |
| p80:c0 | Non-manipulated – Fungus | 0.002 | 0.04 | 0.96 |
| p80:c0 | Tween control – Fungus | -0.03 | -0.6 | 0.96 |
| p80:c0 | Non-viable spores – Fungus | 0.08 | 1.71 | 0.25 |
| p64:c16 | Non-manipulated – Fungus | 0.01 | 0.31 | 0.75 |
| p64:c16 | Tween control – Fungus | -0.03 | -0.65 | 0.75 |
| p64:c16 | Non-viable spores – Fungus | 0.11 | 2.18 | 0.08 |
| p40:c40 | Non-manipulated – Fungus | 0.17 | 3.38 | **<0.01** |
| p40:c40 | Tween control – Fungus | 0.004 | 0.08 | 0.93 |
| p40:c40 | Non-viable spores – Fungus | 0.03 | 0.7 | 0.93 |
| p16:c64 | Non-manipulated – Fungus | 0.09 | 1.79 | 0.15 |
| p16:c64 | Tween control – Fungus | 0.09 | 1.76 | 0.15 |
| p16:c64 | Non-viable spores – Fungus | 0.02 | 0.45 | 0.64 |
| p0:c80 | Non-manipulated – Fungus | 0.21 | 4.23 | **<0.001** |
| p0:c80 | Tween control – Fungus | 0.07 | 1.51 | 0.12 |
| p0:c80 | Non-viable spores – Fungus | 0.18 | 3.67 | **<0.001** |

Table S2 Comparisons of the different diets and their effect on the protein amount in spermatophores of *T. molitor* males according to the Least Significant Difference post-hoc test.

| Macronutrient | Diet | Estimate | *t* | p |
| --- | --- | --- | --- | --- |
| Proteins | p80:c0 – p0:c80 | 8.46 | 0.93 | 0.34 |
| Proteins | p64:c16 – p0:c80 | 20.28 | 2.24 | 0.07 |
| Proteins | p40:c40 – p0:c80 | -16.1 | -1.78 | 0.14 |
| Proteins | p16:c64 – p0:c80 | -38.78 | -4.3 | **<0.0001** |

Table S3 Comparisons of the different health states and their effect on the protein amount in spermatophores of *T. molitor* males according to the Least Significant Difference post-hoc test.

| Macronutrient | State of health | Estimate | *t* | P |
| --- | --- | --- | --- | --- |
| Proteins | Non-manipulated – Fungus | -84.13 | -12.38 | **<0.0001** |
| Proteins | Tween control – Fungus | -38.4 | -5.65 | **<0.0001** |
| Proteins | Non-viable spores – Fungus | -68.57 | -10.09 | **<0.0001** |

Table S4 Comparisons of the different diets and their effect on the lipid amount in spermatophores of *T. molitor* males according to the Least Significant Difference post-hoc test.

| Macronutrient | Diet | Estimate | *t* | p |
| --- | --- | --- | --- | --- |
| Lipids | p80:c0 – p0:c80 | -20.4 | -3.47 | **<0.01** |
| Lipids | p64:c16 – p0:c80 | 97.61 | 16.63 | **<0.0001** |
| Lipids | p40:c40 – p0:c80 | 18.05 | 3.07 | **<0.01** |
| Lipids | p16:c64 – p0:c80 | -25.35 | -4.32 | **<0.0001** |

Table S5 Comparisons of the different health states and their effect on the lipid amount in spermatophores of *T. molitor* males according to the Least Significant Difference post-hoc test.

| Macronutrient | State of health | Estimate | *t* | P |
| --- | --- | --- | --- | --- |
| Lipids | Non-manipulated – Fungus | -52.57 | -11.94 | **<0.0001** |
| Lipids | Tween control – Fungus | -12.4 | -2.81 | **<0.01** |
| Lipids | Non-viable spores – Fungus | -72.85 | -16.54 | **<0.0001** |

Table S6 Comparisons of the different diets and their effect on the carbohydrate amount in spermatophores of *T. molitor* males according to the Least Significant Difference post-hoc test.

| Macronutrient | Diet | Estimate | *t* | p |
| --- | --- | --- | --- | --- |
| Carbohydrates | p80:c0 – p0:c80 | 41.48 | 1.82 | 0.13 |
| Carbohydrates | p64:c16 – p0:c80 | 276.97 | 12.19 | **<0.0001** |
| Carbohydrates | p40:c40 – p0:c80 | 180.74 | 7.96 | **<0.01** |
| Carbohydrates | p16:c64 – p0:c80 | -29.72 | -1.3 | 0.19 |

Table S7 Comparisons of the different health states and their effect on the carbohydrate amount in spermatophores of *T. molitor* males according to the Least Significant Difference post-hoc test.

| Macronutrient | State of health | Estimate | *t* | P |
| --- | --- | --- | --- | --- |
| Carbohydrates | Non-manipulated – Fungus | -361.63 | -20.84 | **<0.0001** |
| Carbohydrates | Tween control – Fungus | -230.13 | -13.26 | **<0.0001** |
| Carbohydrates | Non-viable spores – Fungus | -250.25 | -14.42 | **<0.0001** |

Table S8 Comparisons of the diet treatment, and its effect on total nutrients amount in spermatophores of *T. molitor* males according to the Least Significant Difference post-hoc test.

| State of health | Diet | Estimate | *t* | P |
| --- | --- | --- | --- | --- |
| Non-manipulated | p80:c0 – p0:c80 | 116.65 | 0.94 | 0.84 |
| Non-manipulated | p64:c16 – p0:c80 | 372.66 | 3.01 | **<0.05** |
| Non-manipulated | p40:c0 – p0:c80 | 97.28 | 0.78 | 0.84 |
| Non-manipulated | p16:c64 – p0:c80 | -24.01 | -0.19 | 0.84 |
| Tween control | p80:c0 – p0:c80 | 125.6 | 1.01 | 0.3 |
| Tween control | p64:c16 – p0:c80 | 394.02 | 3.19 | **<0.01** |
| Tween control | p40:c0 – p0:c80 | 383.89 | 3.1 | **<0.01** |
| Tween control | p16:c64 – p0:c80 | -242.61 | -1.96 | 0.09 |
| Non-viable spores | p80:c0 – p0:c80 | 39.31 | 0.31 | 0.75 |
| Non-viable spores | p64:c16 – p0:c80 | 504.49 | 4.08 | **<0.001** |
| Non-viable spores | p40:c0 – p0:c80 | 294 | 2.38 | 0.05 |
| Non-viable spores | p16:c64 – p0:c80 | 240.02 | 1.94 | 0.1 |
| Fungus | p80:c0 – p0:c80 | -163.4 | -1.32 | 0.37 |
| Fungus | p64:c16 – p0:c80 | 308.3 | 2.49 | **<0.05** |
| Fungus | p40:c0 – p0:c80 | -44.39 | -0.36 | 0.71 |
| Fungus | p16:c64 – p0:c80 | -348.87 | -2.82 | **<0.05** |

Table S9 Comparisons of state of health treatment and its effect on total nutrients amount in spermatophores of *T. molitor* males according to the Least Significant Difference post-hoc test.

| Diet | State of health | Estimate | *t* | P |
| --- | --- | --- | --- | --- |
| p80:c0 | Non-manipulated – Fungus | -380.48 | -3.08 | **<0.01** |
| p80:c0 | Tween control – Fungus | -173.78 | -1.4 | 0.15 |
| p80:c0 | Non-viable spores – Fungus | -454.2 | -3.67 | **<0.001** |
| p64:c16 | Non-manipulated – Fungus | -596.18 | -4.82 | **<0.0001** |
| p64:c16 | Tween control – Fungus | -377.08 | -3.05 | **<0.01** |
| p64:c16 | Non-viable spores – Fungus | -460.74 | -3.73 | **<0.001** |
| p40:c40 | Non-manipulated – Fungus | -518.86 | -4.2 | **<0.001** |
| p40:c40 | Tween control – Fungus | -34.5 | -0.27 | 0.78 |
| p40:c40 | Non-viable spores – Fungus | -318.52 | -2.57 | **<0.05** |
| p16:c64 | Non-manipulated – Fungus | -335.68 | -2.71 | **<0.05** |
| p16:c64 | Tween control – Fungus | -356.54 | -2.88 | **<0.05** |
| p16:c64 | Non-viable spores – Fungus | -68.02 | -0.55 | 0.58 |
| p0:c80 | Non-manipulated – Fungus | -660.54 | -5.34 | **<0.0001** |
| p0:c80 | Tween control – Fungus | -462.79 | -3.74 | **<0.001** |
| p0:c80 | Non-viable spores – Fungus | -656.92 | -5.32 | **<0.0001** |
