# Supplementary material for: Dietary macronutrient balance and fungal infection as drivers of spermatophore quality in the mealworm beetle
Source: Curr Res Insect Sci. 2021 Jan 16;1:100009. doi: 10.1016/j.cris.2021.100009 (PMC9387488; doi:10.1016/j.cris.2021.100009)
Supplement: Supplementary file 2 [file mmc2.pdf]

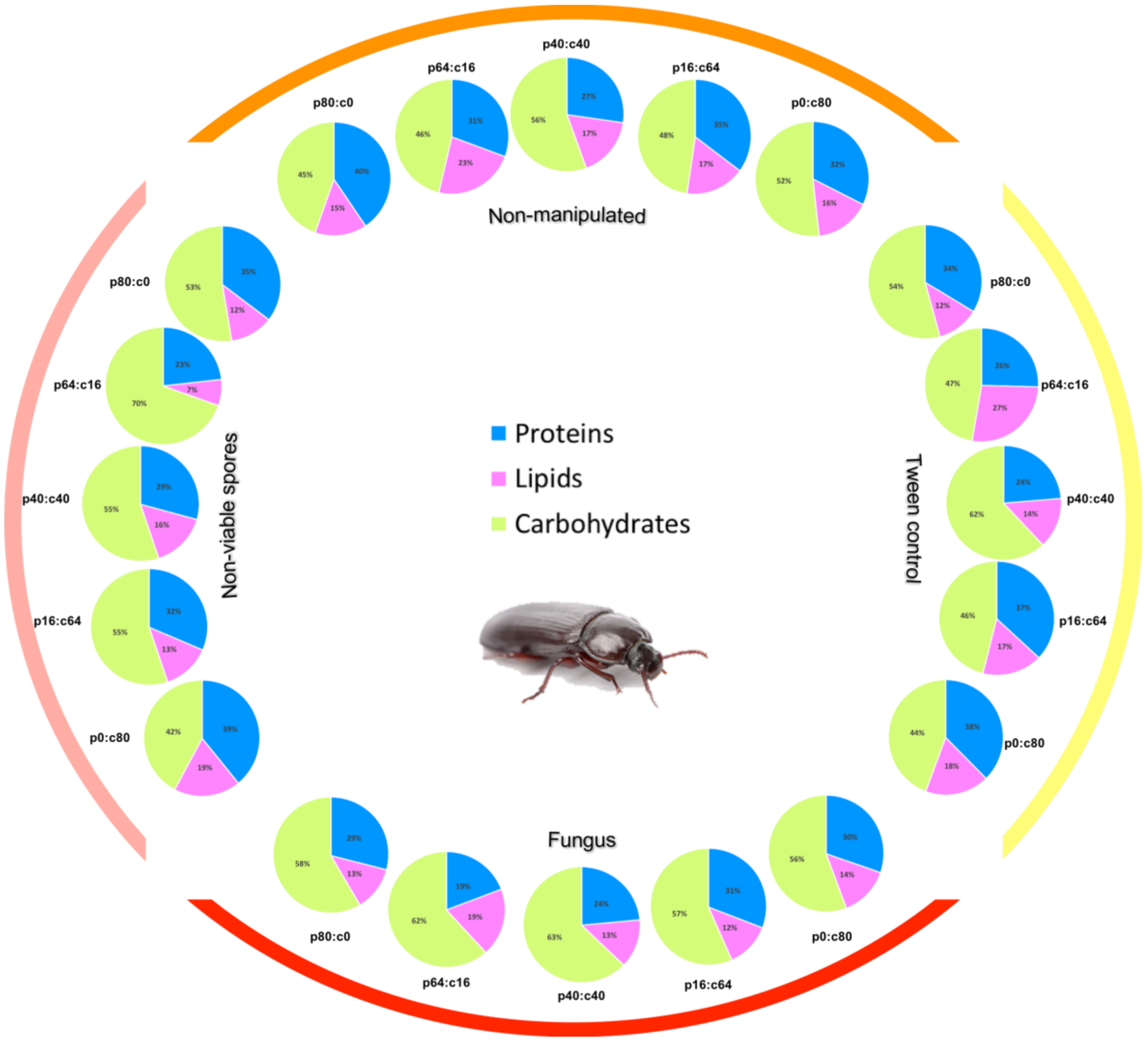

Supplementary material Figure 1. Visual comparisons of relative carbohydrate, lipid and protein allocation to spermatophore following changing ratios of protein:carbohydrate (p:c) in the male diet and manipulation of health status (fungus: fungal challenged males; and controls: Tween control, non-viable spores, and non-manipulated) in *Tenebrio molitor* males.
